# The Effect of Age on Post‐Stroke Language Outcomes

**DOI:** 10.1155/jare/7040010

**Published:** 2026-06-23

**Authors:** Sophie M. Roberts, Rachel M. Bruce, Thomas M. H. Hope, Storm Anderson, Hayley Woodgate, Kate Ledingham, Alexander P. Leff, David W. Green, Cathy J. Price

**Affiliations:** ^1^ Department of Imaging Neuroscience, University College London, London, UK, ucl.ac.uk; ^2^ Institute of Cognitive Neuroscience, University College London, London, UK, ucl.ac.uk; ^3^ Department of Brain Repair and Rehabilitation, University College London, London, UK, ucl.ac.uk; ^4^ Department of Experimental Psychology, University College London, London, UK, ucl.ac.uk

## Abstract

**Introduction:**

This study investigates how chronological age at stroke onset affects post‐stroke language abilities and recovery. Using cross‐sectional and retrospective data, we investigated (A) which language tasks are sensitive to age; (B) whether any age‐related effects are modified by factors such as lesion size, initial severity and education and (C) whether age influences recovery from aphasia.

**Methods:**

Language abilities were assessed in 749 participants using (i) the Comprehensive Aphasia Test (CAT) at a single time point and (ii) self‐reported measures of speaking, understanding, reading and writing ability both at 1 week and 1 year post‐stroke. The outcomes investigated, using multiple linear regression, were (1) CAT language scores and (2) self‐reported recovery scores at 1 year post‐stroke. Lesion size, initial severity, sex, handedness, pre‐stroke education and time post‐stroke were controlled. Bayesian statistics assessed the strength of evidence for observed effects.

**Results:**

(A) Irrespective of lesion or symptom severity, older age at stroke onset was associated with worse performance across multiple language domains, including overall language ability, nonword and word repetition, semantic and letter fluency, object naming and comprehension of spoken and written words and sentences. Effect sizes were strongest for nonword repetition (*β* = −0.258) and weakest for written word comprehension (*β* = −0.061). (B) The disadvantage of older age was heightened in participants with the largest lesions and most severe initial aphasia, but (C) there was no evidence that older age hindered language recovery in any subgroup.

**Conclusion:**

Older age at stroke onset significantly worsened language outcomes, highlighting the sensitivity of aphasia assessments to age‐related differences in post‐stroke language abilities. This disadvantage was greatest in participants with both large lesions and severe initial symptoms. Considering age when interpreting assessment scores would optimise diagnosis and prognostic accuracy.

## 1. Introduction

This study investigates how chronological age at stroke onset influences post‐stroke language ability and recovery. Specifically, it investigates (A) which language task scores are most sensitive to age at stroke, (B) whether these age‐related effects are modified by key variables such as stroke severity and (C) how age influences post‐stroke language recovery. Addressing these questions is a high research priority because the number of people living into older age with language difficulties (aphasia) is likely to increase due to a combination of the decreasing average age at stroke [1] and longer survival as a result of better treatments [2]. A better understanding of how age affects aphasia outcomes across the lifespan has the potential to refine prognostic models, offering more personalised and accurate information about expected language abilities and likely recovery.

Age effects on language have been observed across various languages in neurotypical adults, with their impact varying with the type of language function tested. For example, lexical retrieval tasks, including naming and verbal fluency, appear to be more vulnerable to ageing than comprehension and semantic knowledge [3, 4]. Additionally, age‐related effects may depend on linguistic factors like syntactic complexity in comprehension [5, 6], and whether a fluency task taps semantic or phonemic retrieval [7–10]. Detecting age‐related effects may also depend on the type of analysis used. For example, nonlinear models may outperform linear models at capturing midlife language expansion and later‐life decline [11, 12]. Age effects may also depend on socioeconomic variables: for example, in Spanish adults, older age was more disadvantageous for those who undertook less education [13].

In healthy adults, normal ageing effects on language performance likely reflect several underlying age‐related processes [4]. These include biological ageing through brain atrophy and subsequent changes to cerebrovascular function, sensory declines like hearing loss, which can impair speech comprehension; visual loss, which affects reading and visuo‐perceptual abilities [14]; reduced motor control, which could affect speech production and cognitive changes such as slower processing speed; diminished working memory and reduced attention, which could hinder the ability to follow and participate in a conversation [15–17]. Additionally, frailty and multiple comorbidities could limit physical capabilities and functional independence [18], reducing opportunities to practise language through social interactions.

Amongst the stroke population, older age is also associated with (i) more comorbid conditions and risk factors, which inhibit rehabilitation and recovery [19, 20]; (ii) reduced neuroplasticity, which is essential for post‐stroke neural reorganisation [21] and (iii) less grey and white matter volume [22], which reduces the integrity of brain networks and increases biological ‘brain age’ relative to chronological age. Biological brain age, estimated from reduced cortical thickness and white matter, can be accelerated in individuals with neurodegeneration [23, 24], excessive alcohol use [25], higher visceral fat levels [26], physio‐cognitive decline [27] and poorer cognitive function after stroke [28]. These associations are not necessarily causal and should be interpreted carefully [29], but the key point here is that the effects of chronological age after stroke may depend on numerous factors, such as brain integrity and cognitive function.

The effect of age at stroke onset on aphasia outcomes and recovery remains poorly understood. A search of PubMed, using terms such as ‘age at stroke’ and ‘post‐stroke aphasia’ identified 12 studies of aphasia outcome and 17 studies of aphasia recovery. A summary of these studies is provided in Tables S1–S3; organised according to their focus (aphasia outcomes or recovery), their conclusions (significant or no significant effects of age) and key study characteristics such as sample size, aphasia outcome measures, effect sizes (when these were reported) and whether major covariates such as lesion factors, demographics, cognition, clinical severity and time post‐stroke were controlled. The effects of age were very inconsistent across studies. In the 12 studies of aphasia outcome, 6 reported a benefit of younger age, whereas 5 reported no relationship with age. The remaining study, by Johnson et al. [30], found an effect of age on aphasia outcome only after controlling for demographic factors and not when health and lesion factors were also controlled. In addition, this study found a significant correlation between age and white matter integrity, suggesting that, as observed in neurotypical people, the effect of age may be related to the integrity of residual brain networks. Inconsistency was also observed in the 18 studies of aphasia recovery: 8 reported either a significant relationship or trend, where older age resulted in worse recovery, while 10 found no relationship between age and recovery.

Table S1 also demonstrates several methodological differences that might explain the inconsistent findings of age effects in aphasia outcomes and recovery: (i) sample sizes ranged from 14 [31] to >4000 [32]; (ii) recovery time frames ranged from subacute [33] to chronic [34]; (iii) outcome measures differed, from broad measures of aphasia severity [35] to more specific tasks, such as Naming, which is more sensitive to ageing [36]; (iv) therapy was provided in some [37] but not all [38] studies; (v) the influence of brain age, beyond chronological age, was only considered in two studies [30, 39] and (vi) other sources of variance were inconsistently controlled. Variables which are often, but not always, considered include: sex, initial aphasia severity, lesion size, stroke type, cognitive abilities, and education, among many others. Differing combinations of variables across studies may explain their varying sensitivity to age effects.

The current study aimed to better understand the variables that determine the effect of age on language performance. To do this, we drew on cross‐sectional data from a wide range of language tasks administered uniformly to a large and demographically diverse sample of stroke survivors with varying degrees of stroke and symptom severity. This approach allows us to examine ageing‐related changes in language task performance and how they are shaped by stroke and aphasia. Based on studies of healthy ageing, our overarching hypothesis was that a disadvantage of older age would be observed on post‐stroke language outcomes, and this effect would vary with task and other variables, investigated in questions A and B. With respect to which language tasks are most sensitive to age (Question A), our hypothesis was that older age at stroke would result in poorer performance on tasks that use word retrieval, such as Naming, Picture description and Fluency [40]. For comprehension tasks, there were two opposing possibilities. An effect of older age could be observed if working memory is reduced amongst older adults. In contrast, if older adults are able to compensate for memory impairments using their linguistic experience gained over the lifetime, no effect of age would be observed on these tasks [5].

With respect to whether the effect of age depended on other variables (Question B), we investigated alternative hypotheses. The first was that the disadvantage of older age would be more pronounced in participants who experienced the most severe initial aphasia because aphasia impairs the encoding and consolidation processes needed for word relearning [41–44]. The second possibility is that age effects will not be detected in patients with large strokes because they have limited neural capacity, which is necessary for recovery irrespective of age.

With respect to whether age at stroke onset influenced recovery (Question C), our hypothesis was that older age would hinder language recovery, after controlling for a wider range of variables than previous studies.

The work in this manuscript was conducted as part of a PhD thesis [45]. This is available on University College London’s open‐access repository: https://discovery.ucl.ac.uk/.

## 2. Methods

The study was approved by the London Queen Square Research Ethics Committee (study reference 13/LO/1515). All participants gave written informed consent (or assent via a consultee) to participate.

### 2.1. Participant Selection

Participants were selected from the Predicting Language Outcome and Recovery After Stroke (PLORAS) database [46]. The database holds demographic, behavioural and lesion data for over 3000 stroke patients, who are recruited from various locations around the United Kingdom, including via the National Institute for Health and Care Research (NIHR) Research Delivery Network, stroke groups and conferences, and word‐of‐mouth recommendations. Participants were eligible for this study if they (1) had a clinically diagnosed stroke in either hemisphere (including the cerebellum, but excluding the brainstem), (2) were native English speakers, (3) had a research MRI brain scan and complete language assessment scores and (4) had no other neurological disorders that might affect their speech and language abilities. A total of 1129 participants met these criteria. Participants were then excluded if (a) their language abilities 1 week post‐stroke were unknown (*n* = 357), (b) their education was unknown or outlying (*n* = 11) and (c) they reported normal speech 1 week post‐stroke, but scored in the aphasic range on Naming or Picture Description when assessed (typically well into the chronic stage of recovery)—suggesting an unreliable self‐report (*n* = 12). These criteria identified the final sample of 749 participants (see Table 1). Participants with missing data for some covariates were excluded from analyses that included those variables; see the description of the statistical analyses below for more details.

**TABLE 1:** Participant characteristics.

Number		749
Age at stroke onset (years)	Mean (SD)	57 (13)
	Range	17 to 99

Sex	N male (%)	518 (69)
	N female (%)	231 (31)

Initial speaking severity	Severe (%)	243 (32)
	Moderate (%)	118 (16)
	Mild (%)	242 (32)
	Normal (%)	146 (19)

Years of education after age 14	Mean (SD)	5 (3)
	Range	0 to 13

Years post‐stroke of CAT	Mean (SD)	4 (5)
	Range	0 to 42

Years post‐stroke of scan	Mean (SD)	3.5 (4)
	Range	0 to 42

Lesioned hemisphere	Left (%)	383 (51)
	Right (%)	201 (27)
	Both (%)	134 (18)
	Undetected (%)	31 (4)

Left hemisphere lesion size (cm^3^)	Mean (SD)	31 (58)
	Range	0 to 428

Right hemisphere lesion size (cm^3^)	Mean (SD)	17 (46)
	Range	0 to 357

Pre‐stroke handedness	Right (%)	648 (87)
	Left (%)	77 (10)
	Ambidextrous (%)	24 (3)

Developmental dyslexia status	Formal diagnosis (%)	14 (2)
	None (%)	425 (57)
	Difficulties relative to peers^a^ (%)	52 (7)
	Unknown (%)	258 (34)

^a^‘Difficulties relative to peers’ is reported when participants had reading or spelling difficulties compared to their peers that may indicate developmental dyslexia, but did not receive a formal diagnosis (usually because they were not assessed for dyslexia). For statistical analyses, these participants were grouped with those whose dyslexia status was ‘unknown’.

### 2.2. Age at Stroke Onset and Other Demographic Variables

Age at stroke onset was calculated from participants’ date of birth and date of stroke, both of which were recorded in demographic questionnaires. These questionnaires also captured self‐reported information for biological sex, pre‐stroke handedness, developmental dyslexia status and amount of pre‐stroke formal education (measured as the number of years completed after the age of 14, because this was the least amount of education received in the sample). Age at test was not included in the models but is represented indirectly as the sum of age at stroke and time post‐stroke.

### 2.3. Initial Aphasia Severity

Each participant was assigned an initial severity rating, derived from an in‐house retrospective participant‐reported outcome measure of four language functions (speaking, understanding, reading and writing) at 1 week post‐stroke. Each function was rated on an ordinal scale with four levels of severity: Severe (unable to produce/comprehend any speech/writing, or only able to produce automatic speech), Moderate (able to produce/comprehend single words and phrases), Mild (able to produce/comprehend short sentences) and Normal. Registered speech and language therapists supported participants to provide an accurate report of their difficulties; for example, differentiating fluent from nonfluent aphasia, or dysarthria versus aphasia. If participants were not able to retrospectively rate their language abilities, a carer provided their retrospective ratings of their language instead. The ratings were repeated for each language function. A participant could therefore be assigned different initial severity ratings for different language functions (if, for example, they had speaking difficulties, but intact comprehension). Participants who rated themselves as Severe at 1 week post‐stroke reported being conscious and medically capable of attempting to speak—but unable to produce any words, due to aphasia (and/or dysarthria/apraxia). These participants were included in the study and are distinct from those who were excluded because they reported being incapable of attempting to speak (e.g., unwell, in a coma, ventilated) and therefore unable to provide retrospective ratings of their initial abilities.

### 2.4. Lesion Data

High‐resolution (1 mm × 1 mm × 1 mm), whole brain T1‐weighted structural brain images were acquired for all participants on research‐dedicated scanners at the Wellcome Centre for Human Neuroimaging in UCL’s Department of Imaging Neuroscience and the Birkbeck‐UCL Centre for Neuroimaging. The MRI scanners used were all from Siemens Healthcare (Erlangen, Germany): 497 were imaged on a 3T Trio (53 of which were after Prisma upgrade), 11 were imaged on a 3T Allegra, 176 were imaged on a 1.5T Avanto and 65 were imaged on a 1.5T Sonata. For the majority (82%) of the sample, brain scans were obtained on the same day as the language assessments (see Table 1). Where there was a delay between scan and assessment, 4% were obtained within 1 month of each other, and 3% within 1 year. The remaining 11% were beyond 1 year.

Using standard procedures within SPM software (https://www.fil.ion.ucl.ac.uk/spm/), running in MATLAB environment (2018a Mathworks, Sherbon, MA, USA), each T1‐weighted image was spatially normalised (to the MNI template) and converted into a quantitative assessment of structural abnormality that is independent of the scanner used [47]. At each voxel, this ‘fuzzy’ lesion image encodes the degree of abnormality on a continuous scale from 0 (completely normal) to 1 (completely abnormal) relative to normative data from a sample of 64 neurologically intact controls, reported in Seghier et al. [47]. The 64 controls were aged from 21 to 75 years, thus ensuring that any normal ageing‐related structural changes, such as enlarged ventricles, were not erroneously classified as lesions in the patients, but instead represented and identified as ‘normal’ (for the age range). To delineate the lesions and estimate lesion volume, each fuzzy lesion image was thresholded into a ‘binary’ lesion image (i.e., presence or absence of a lesion, at each voxel). The abnormality threshold used was 0.3 (U value, on the 0 to 1 scale described above), as recommended in Seghier et al. [47], after optimisation from data collected on the scanners used for image acquisition in the current study. Separate lesion size estimates were made for left and right hemisphere lesion size.

All scans and lesion identifications were then visually inspected, as recommended by Seghier et al. [47]. The only systematic mis‐identification we observed was a false‐positive cluster in the brainstem; therefore, the brainstem was removed from all lesion images before calculating lesion size. Beyond this, no further automated lesion markings were removed, reduced or added. Although the remaining automated lesion images did not always match the visual interpretation exactly, they were left unchanged for the analyses presented in this paper. Despite this imprecision, the automated lesion identification outputs, showed by far the strongest associations with language outcomes. This careful review process minimised the risk of misidentifying age‐related changes as lesions, thereby enhancing the accuracy of our lesion identification and interpretation.

### 2.5. Language Outcomes

All participants were assessed with a standardised language and cognitive assessment—the Comprehensive Aphasia Test (CAT) [48]. The timing of CAT assessment was different for each participant, ranging from 17 days to 42 years post‐stroke. The vast majority of assessments were obtained after the one‐year post‐stroke timepoint had passed, and a minority (*n* = 65, 9% of the sample) were obtained within 6 months of stroke onset. To compare performance on each of the 27 tasks (with varying demands and difficulty), raw scores are converted through a nonlinear transformation into T‐scores, using a conversion table in the CAT manual. The transformation (i) converts raw scores to percentile ranks based on an aphasic reference sample (113 individuals; 266 test scores), (ii) maps these ranks to Z‐scores using the inverse normal transformation and (iii) converts *Z* scores to T‐scores using *T* = 10*Z* + 50 to yield a mean of 50 and a standard deviation of 10. The outputs therefore represent how well the participant performed relative to an independent sample of patients with aphasia, without normalising to age, education, gender or other variables. Lower scores indicate poorer performance. The current study focused on 19 of these tasks, and 6 ‘summary’ scores that integrate scores from tasks that assess the main language domains:

(1) Naming: object naming, action naming and verbal fluency (semantic and letter).
(2) Repetition: repetition of simple and complex words, function words and nonwords.
(3) Reading: reading aloud simple and complex words, function words and nonwords.
(4) Spoken picture description: number of information‐carrying words produced in 1 min, grammatical well‐formedness, syntactic variety and speech fluency.
(5) Spoken comprehension: from spoken word‐to‐picture matching, sentence‐to‐picture matching and paragraph comprehension.
(6) Written comprehension: written word‐to‐picture matching and sentence‐to‐picture matching.

In addition, these six summary scores were averaged to construct an ‘overall language ability’ score, as in Winans‐Mitrik et al. [49]. ‘Overall language ability’ was analysed as a first step, and served as a precursor for more detailed subsequent analyses on specific language skills (see Statistical Analyses section below). Writing ability was not investigated because stroke‐related motor impairments were not formally assessed, which could impact writing ability in addition to age‐related motor difficulties.

**FIGURE 1:**
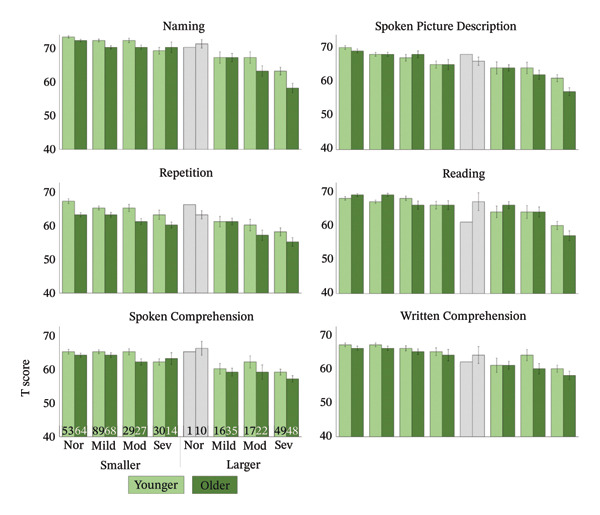
The effect of age in 572 participants, grouped according to left hemisphere lesion size and initial symptom severity, on 6 summary scores from the CAT. Legend: T‐scores for each of the six CAT summary scores, grouped by age, initial speaking severity and left hemisphere lesion size. The colours indicate younger (light green; ≤ 57.88 years) and older (dark green; ≥ 57.93 years), the threshold for which was set using the full sample median. Grey bars indicate where the group comprised 10 or fewer participants (i.e., not well‐sampled). Nor, Mild, Mod and Sev refer to the four self‐reported severity levels (Normal, mild aphasia, moderate aphasia or severe aphasia. ‘Smaller’ (first 8 bars) refers to participants with smaller left hemisphere lesions (0–3 cm^3^). ‘Larger’ (last 8 bars) refers to participants with larger left hemisphere lesions (more than 9 cm^3^). The number of participants in each group is shown in the plots for spoken comprehension (bottom left) and is the same for all summary scores. The total number of participants illustrated is 572, after 177 were excluded to match groups for left hemisphere lesion size and time post‐stroke (see Methods section). Score thresholds indicating aphasia: < 63 for Naming, < 61 for Spoken picture description < 60 for Repetition, < 61 for Reading, < 57 for Spoken comprehension, < 60 for Written comprehension.

For all tasks and summary scores, T‐scores were analysed. The exception was semantic and letter fluency, for which raw scores were analysed because T‐scores were not available for the specific conditions. An aphasic/normal threshold is available in the CAT manual for each language summary skill. This threshold indicates whether a T‐score falls into the aphasic or the normal range. For example, for Naming, T‐scores of 62 and lower indicate aphasia, and 63 and above indicate the normal range. The thresholds are given in the legend of Figure 1.

### 2.6. Statistical Analyses

Analyses were conducted in IBM SPSS Statistics (version 28) to address the three research questions (A, B and C).

#### 2.6.1. Which Language Tasks Are Most Sensitive to Age at Stroke Onset?

This question was approached using three steps. (1) Is there a main effect of age on overall language ability score, relative to initial ability? (2) Which summary scores (e.g., naming, repetition, etc.) drive this effect? (3) Which individual tasks drive effects of age in the summary scores? As there is only one statistical analysis in the first step, correction for multiple comparisons is not needed. The second and third steps involve 6 and 24 different analyses (one for each summary and individual task score). If the main effect in step one is significant, the second and third steps can be treated as exploratory or descriptive, and therefore do not require correction for multiple comparisons. However, the table legends also note which summary and task scores would survive a correction for multiple comparisons if they were considered independently.

Each of the analyses described above was conducted using multiple linear regression. The following covariates were included in all analyses to factor out the effects of: education, left hemisphere lesion size, right hemisphere lesion size, sex, pre‐stroke handedness and developmental dyslexia status. Time post‐stroke of CAT was included in all analyses to control for the varying levels of recovery that were expected to occur during this period. Additional covariates depended on the task scores being considered. For example: initial speaking ability was included in the analyses of speaking scores; initial understanding ability was included in the analyses of auditory comprehension scores, initial reading ability was included in the analyses of reading scores, vision status was considered for tasks that used visual stimuli, hearing status was included for tasks that used auditory stimuli, and semantic memory scores were included for tasks that use picture or conceptual stimuli. See Table 2 for the covariates selected for each task analysis. The ‘enter’ regression method was used, which forces all covariates into the model irrespective of their effect—this was to avoid missing any unexpected but potentially significant effects. Each analysis had a different sample size, because participants were excluded from an analysis if they were missing data for one of the required covariates. For example, 173 participants were missing self‐reported reading or understanding data, because participants either could not remember or had not attempted these skills 1 week post‐stroke. Six more had missing hearing or vision data, and four more had missing CAT scores for various tasks. The sample size for each analysis is reported in Table 3, with the impact of sample size variations considered in the Discussion section.

**TABLE 2:** The additional covariates factored out of each CAT analysis.

	Init speak	Init read	Init und	Obj recog	Vision	Hear
Overall language ability	+	+	+	+	+	+
Naming:	+	−	−	+	+	−
Semantic fluency	+	−	−	+	−	−
Letter fluency	+	−	−	+	−	−
Object naming	+	−	−	+	+	−
Action naming	+	−	−	+	+	−
Repetition:	+	−	−	+	−	+
Word repetition	+	−	−	+	−	+
Complex word repetition	+	−	−	+	−	+
Nonword repetition	+	−	−	−	−	+
Digit span	+	−	−	−	−	+
Sentence repetition	+	−	−	+	−	+
Reading:	−	+	−	+	+	−
Reading words	−	+	−	+	+	−
Reading complex words	−	+	−	+	+	−
Reading function words	−	+	−	+	+	−
Reading nonwords	−	+	−	−	+	−
Spoken picture description:	+	−	−	+	+	−
Total number of words	+	−	−	+	+	−
Number of appropriate ICWs	+	−	−	+	+	−
Number of inappropriate ICWs	+	−	−	+	+	−
Grammatical well‐formedness	+	−	−	+	+	−
Syntactic variety	+	−	−	+	+	−
Speed	+	−	−	+	+	−
Spoken comprehension:	−	−	+	+	+	+
Spoken word‐to‐picture	−	−	+	+	+	+
Spoken sentence‐to‐picture	−	−	+	+	+	+
Spoken paragraph	−	−	+	+	−	+
Written comprehension:	−	+	−	+	+	−
Written word‐to‐picture	−	+	−	+	+	−
Written sentence‐to‐picture	−	+	−	+	+	−

*Note:* Init speak = initial speaking severity; Init read = initial reading severity; Init und = initial understanding severity; Obj recog = object recognition; Vision = vision status; Hear = hearing status. Each analysis also factored out: sex, handedness, education, time post‐stroke of CAT, left hemisphere lesion size, right hemisphere lesion size, and dyslexia status. ICWs = information carrying words.

**TABLE 3:** The effects of age on CAT assessment scores.

Analysis type		Multiple linear regression			Bayesian regression	
Outcome measure	*N*	*R* ^2^	*p* value	*β*	Log BF	Evidence strength
Overall language ability	569	0.632	< 0.001	−0.125	3.04	Decisive
Naming:	741	0.580	< 0.001^∗^	−0.149	4.47	Decisive
Semantic fluency	748	0.423	< 0.001^∗^	−0.234	9.16	Decisive
Letter fluency	748	0.384	< 0.001^∗^	−0.119	1.14	Strong
Object naming	741	0.473	< 0.001^∗^	−0.106	0.91	Substantial
Action naming	741	0.455	ns	−0.049		
Picture description:	741	0.516	0.008	−0.074	−0.22	—
Total number of words	741	0.415	ns	−0.055		
Number of appropriate ICWs	741	0.441	0.026	−0.067	−0.61	—
Number of inappropriate ICWs	741	0.134	0.003	0.111	0.10	Anecdotal
Grammatical well‐formedness	741	0.433	ns	−0.002		
Syntactic variety	741	0.418	ns	−0.005		
Speed	741	0.438	ns	−0.055		
Repetition:	743	0.478	< 0.001^∗^	−0.169	4.59	Decisive
Word repetition	743	0.380	< 0.001^∗^	−0.201	5.68	Decisive
Complex word repetition	743	0.387	0.008	−0.083	−0.25	—
Nonword repetition	743	0.342	< 0.001^∗^	−0.258	9.81	Decisive
Digit span	744	0.391	ns	−0.043		
Sentence repetition	743	0.432	ns	0.027		
Reading:	572	0.444	ns	0.029		
Reading words	573	0.435	ns	0.027		
Reading complex words	572	0.431	ns	0.027		
Reading function words	572	0.188	ns	−0.015		
Reading nonwords	572	0.394	ns	−0.007		
Spoken comprehension:	709	0.388	< 0.001^∗^	−0.167	3.32	Decisive
Spoken word‐to‐picture	711	0.272	< 0.001^∗^	−0.164	2.39	Decisive
Spoken sentence‐to‐picture	710	0.400	< 0.001^∗^	−0.140	1.97	Very strong
Spoken paragraph	710	0.118	0.014	−0.096	−0.40	—
Written comprehension:	573	0.525	0.001^∗^	−0.099	0.49	Anecdotal
Written word‐to‐picture	574	0.339	ns	0.061		
Written sentence‐to‐picture	573	0.526	< 0.001^∗^	−0.151	3.01	Decisive

*Note:* Effects of age on overall language ability, and summary and individual task scores from the CAT, using (i) multiple linear regression and (ii) Bayesian regression, when other clinical and demographic variables were factored out. The full sample includes 749 participants, but the number in each analysis (*N*) varies due to missing data for certain covariates. ICWs = Information‐carrying words. ns = not significant at a threshold of *p* = 0.05. BF = Bayes factor: Positive BF supports an effect of age. Evidence strength: > 2 = decisive; 1.5–2 = very strong; 1–1.5 = strong; 0.5–1 = substantial; 0–0.5 = anecdotal [50]. Negative BF refutes the effect (empty cells indicate negative BF and nonsignificant *p* value).

^∗^Significant after correction for multiple comparisons (= 6 summary scores; 24 tasks/subscores).

Bayesian statistics were employed to further validate the results by estimating the strength of evidence for or against age effects on each language measure. This involved conducting a multiple linear regression for each outcome with all the relevant covariates except age, to obtain standardised residuals that were then regressed against age. The resulting Bayes Factors were converted into Log BF, which balanced the scale around 1. Log BF values between 0 and 1 signify negative evidence while values greater than 1 signify positive evidence. This linearly represents the ratio of evidence for either hypothesis and simplifies interpretation. The strength of evidence can also range from ‘anecdotal’ to ‘decisive’ [50]; see Table 3 legend for categories of evidence strength.

#### 2.6.2. How Does the Effect of Age at Stroke Onset Depend on Other Variables?

Moderator analyses were conducted to investigate potential interactions between age and five other variables of interest. The interaction terms were created by multiplying ‘age at stroke onset’ with each variable: left hemisphere lesion size, right hemisphere lesion size, initial severity, education amount and time post‐stroke. Two‐way interaction terms were added into a second block of the model; three‐way interaction terms were added into a third block of the model (along with the relevant two‐way interaction terms) and four‐way interaction terms were added into a fourth block of the model (along with the relevant two‐way and three‐way interaction terms). The first block of the model contained the same covariates as the main analyses. Due to the complexities of investigating and interpreting higher‐order interactions, we limited our approach to only investigating three‐ and four‐way interactions with variables that showed a significant two‐way interaction with age. The two‐way and three‐way interaction analyses included 13–16 terms each, and, based on a guideline of 37 participants per term [51], meant our sample size of 749 was sufficient to support these models. Only the four‐way interaction analysis, which contained 23 terms, was at risk of having too few participants. To mitigate this potentially unstable model, we also supplemented the analysis with post hoc analyses and plots to illustrate the complex higher‐order interactions, described below. These moderator analyses were conducted first for overall language ability, then repeated as post hoc tests for the six summary scores.

Post hoc analyses of significant interactions were conducted after creating 16 subgroups of participants with four levels of initial severity (Normal, Mild, Moderate, Severe), two lesion sizes (larger versus smaller than the median of the full sample = 3.34 cm^3^) and two age categories (older and younger than the median of the full sample = 57.9 years). To ensure that the older and younger groups were matched for lesion size and time post‐stroke, we excluded: 169 participants (85 younger; 84 older) with left hemisphere lesions sized > 100 cm^3^ and < 8.5 cm^3^, and 8 participants (6 younger; 2 older) who were assessed many years post‐stroke (16–42 years). Nevertheless, it was still not possible to fully match the groups for time post‐stroke. This left 374 participants in the ‘smaller’ groups and 198 participants in the ‘larger’ groups (see Table S4).

**FIGURE 2:**
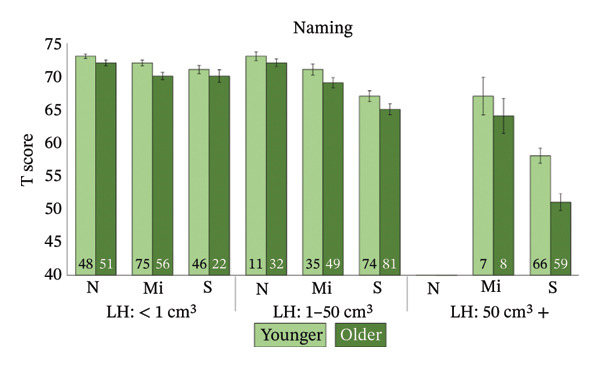
The three‐way interaction between age, left hemisphere lesion size and initial severity on Naming in 720 participants. Legend: The three‐way interaction between age, lesion size and initial severity is illustrated for the average T‐scores for Naming, because it was strongest for this task (see Table 4). Unlike Figure 1: (i) there are three rather than two lesion size groups, and (ii) participants with Moderate initial severity are included in the Severe groups because of small sample sizes. As in Figure 1, younger/older are indicated by light/dark green, and were determined using the median of the full sample. Younger = ≤ 57.88 years; older = ≥ 57.93 years. N, Mi and S refer to three initial severity groups (Normal, Mild, Severe). LH < 1 cm^3^ (first six columns) refers to participants with very small left hemisphere lesions (< 1 cm^3^) and/or larger right hemisphere lesions. LH 1–50 cm^3^ (middle six columns) refers to participants with left hemisphere lesions that were between 1 and 50 cm^3^, irrespective of right hemisphere damage. LH > 50 cm^3^ (last four columns) refers to participants with left hemisphere lesions that were larger than 50 cm^3^, irrespective of right hemisphere damage. Twenty‐nine participants were removed from the full sample to match each pair of age groups for left hemisphere lesion size (see Methods section for details). The numbers at the bottom of the columns show the number in each subgroup. The effect of age was greater in participants who had lesions larger than 50 cm^3^ than lesions that were 1–50 cm^3^—and this effect was greater when participants had severe/moderate initial symptoms than normal or mild initial symptoms.

Post hoc analyses were also conducted after further categorising all 749 participants into three lesion size subgroups: < 1 cm^3^, 1–50 cm^3^ and ≥ 50 cm^3^; and merging Moderate and Severe initial ability categories into one group. This resulted in 18 subgroups (2x age, 3x lesion size and 3x initial severity). To ensure comparable lesion sizes between younger and older participants, 29 individuals (13 younger and 16 older) with the largest and smallest lesions were removed (Table S5 and Figure 2).

#### 2.6.3. Does Age at Stroke Onset Influence the Degree of Recovery?

##### 2.6.3.1. Effects of Age on Cross‐Sectional CAT Scores

An effect of age on recovery would be indicated, across participants, by a significant interaction between age and time post‐stroke (investigated in section B), with post hoc tests showing that the disadvantage of older age grew over time. We also examined the three‐ and four‐way interactions between age, time post‐stroke and (i) left hemisphere lesion size, (ii) initial severity and (iii) education. Note that, as CAT scores were mostly acquired years post‐stroke, the influence of age on early recovery (i.e., within the first year) may not be detected.

##### 2.6.3.2. Effects of Age on Longitudinal Self‐Rated Scores

A proportional ‘recovery score’ was calculated for each participant by dividing [absolute improvement at 1 year post‐stroke] by [improvement potential]. Absolute improvement is the number of ability levels by which a participant increases between 1 week and 1 year [52, 53]. For example, a participant with Severe initial severity who improved to Mild by 1 year improved by 2 severity levels (severe to moderate and moderate to mild). Likewise, a participant with Moderate initial severity who improved to Normal would also have a score of 2 (moderate to mild and mild to normal). In contrast, improvement potential is the total number of severity levels available to increase by. This is maximum for participants with Severe initial symptoms who can move up three levels (severe to moderate, moderate to mild and mild to normal) and minimum for patients with mild initial symptoms who can only move up one level (mild to normal). The proportional recovery score (absolute improvement divided by improvement potential) is the proportion of the total improvement potential. For example, a participant who improved by two out of three possible performance increases would receive a recovery score of 0.66. This proportional recovery score method enables all participants to be analysed together, regardless of their initial severity; however, it still does not distinguish patients with mild from severe initial symptoms if they all make 0% or 100% recovery. The pros and cons of different recovery measures are considered further in the Discussion.

The proportional recovery analysis was repeated for each of the four language skills, resulting in each participant receiving a recovery score for speaking, understanding, reading and writing. The mean of these four recovery scores was also calculated for each participant. Multiple linear regression was used to investigate the relationship between age and recovery score, when left and right hemisphere lesion size, education, sex and handedness were factored out. As with the CAT recovery analyses, we examined two‐ and three‐way interactions between age and (i) left hemisphere, (ii) right hemisphere lesion size and (iii) education to determine whether an effect of age on recovery depended on these other variables.

## 3. Results

### 3.1. Which Language Scores Are Most Sensitive to Age at Stroke Onset?

Across 749 participants, there was a significant, negative effect of age on overall language ability, after factoring out the effect of other variables. As expected, scores were significantly lower for participants who were older than younger at stroke onset. Table 3 and Figure 1 show how this disadvantage of older age was observed for five of the six summary scores: Naming, Spoken Picture Description, Repetition, and Spoken and Written Comprehension (but not Reading aloud). Bayesian evidence was ‘Decisive’ for Naming, Repetition and Spoken Comprehension; and Anecdotal for Written Comprehension.

**FIGURE 3:**
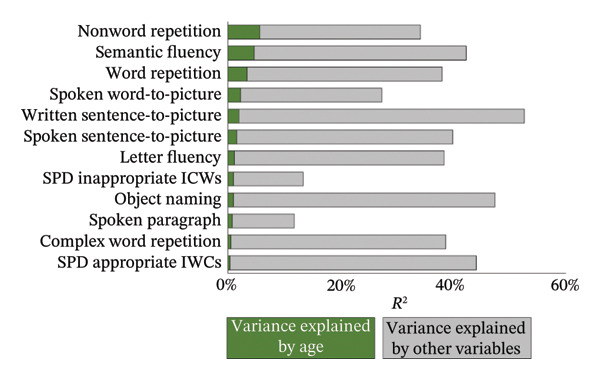
The variance in CAT task scores explained by (i) age at stroke onset and (ii) other variables. Legend: The amount of variance explained by age (green portion of bar) and by all other covariates (grey portion of bar) for the 12 CAT tasks/subscores which were significantly influenced by age at stroke. SPD = spoken picture description; ICW = information‐carrying word. The remaining 12 CAT tasks/subscores (on which there was no significant effect of age) are not shown here.

Table 3 provides (i) the *R* ^2^, *β* and *p* values from separate regression analyses (one for each task that contributed to the summary scores) and (ii) the strength of the evidence from the Bayesian regression, when other variables were factored out of the analyses (for each task that was significant in the regression analyses). Figure 3 shows the amount of variance explained independently by age for each task. The largest amount of variance explained by age was 6% for nonword repetition—which was relatively small, compared to the amount collectively explained by the other covariates, but larger than that explained in prior studies, such as 1.8% on overall language function, by Wilson et al. [54]. After (1) nonword repetition, the effect of age was strongest for: (2) semantic fluency, (3) spoken word‐to‐picture matching, (4) written sentence‐to‐picture matching and (5) the number of inappropriate words produced during spoken picture description (i.e., more inappropriate words were produced by older participants).

### 3.2. How Does the Effect of Age at Stroke Onset Depend on Other Variables?

For all six summary scores, the effect of age was greater for participants with larger (> 9 cm^3^) than smaller (0–3 cm^3^) left hemisphere lesions. This is illustrated in Figure 1 and resulted in significant two‐way interactions between age and lesion size that additionally interacted with initial severity (i.e., a three‐way interaction) for all four speech production scores (Naming, Spoken picture description, Repetition and Reading), but not the comprehension scores. As illustrated in Figures 1 and 2, the effect of age is greater for larger lesions that were associated with severe or moderate initial symptoms than for larger lesions associated with normal or mild initial symptoms.

The effect of age also interacted with years of formal education (see Table 4) with a significant four‐way interaction between age, education, left hemisphere lesion size and initial severity for Naming and Picture Description. These four‐way interactions arose because, in participants with large lesions (over 50 cm^3^), and severe/moderate initial symptoms, the effect of age was enhanced (see Figure 2) and the effect of education was reduced [55].

**TABLE 4:** Interactions between age and other variables on CAT summary scores. Moderator analyses were carried out in 749 participants.

CAT summary score:	*N*	Two‐way: Age &		Three‐way: Age &	Two‐way: Age &	Four‐way: Age &
		LH lesion size	Initial severity	LH lesion size & initial severity	Education	Education, LH lesion size, & initial severity
Overall language ability	569	< 0.001	ns	< 0.001	0.049	ns
Naming	741	< 0.001^∗^	0.001^∗^	< 0.001^∗^	0.038	0.019
Picture description	741	< 0.001^∗^	ns	< 0.001^∗^	ns	0.030
Repetition	743	0.002^∗^	ns	0.005^∗^	0.050	ns
Reading	572	< 0.001^∗^	ns	0.025	0.020	ns
Spoken comprehension	709	< 0.001^∗^	ns	ns	ns	ns
Written comprehension	573	< 0.017	ns	ns	ns	ns

*Note:* *p* values for the two‐ and three‐way interactions between age, left hemisphere lesion size and initial severity, the two‐way interaction between age and education, and the four‐way interaction between age, education, left hemisphere lesion size and initial severity, in 749 participants, for overall language ability, and for each CAT summary score. The number of participants in each analysis (*N*) varies due to missing data for certain covariates. ns = not significant at an uncorrected threshold of *p* = >0.05.

^∗^Significant after correction for multiple comparisons (=6 summary scores). The interactions with education are also reported and discussed in Roberts et al. [55].

### 3.3. The Effect of Age at Stroke Onset on the Degree of Recovery

#### 3.3.1. CAT Scores

Cross‐sectionally, evidence of recovery was observed, as a significant and positive improvement over time on overall language ability (*p* = 0.002, *β* = 0.088), when controlling for all other factors, including the initial severity of symptoms. However, there were no significant interactions with age after correction for multiple comparisons.

We also explored whether log‐transforming time post‐stroke would capture more variance and reveal asymptotic recovery across time, as observed previously in patients tested in the first year post‐stroke [54] and beyond a year [56]. For all seven of our task summary scores, the log‐transformed model performed worse, with lower *R* ^2^ values, effect sizes and significance. This likely reflects the composition of our sample, as most participants (*n* = 411, 55%) were assessed more than 2 years post‐stroke, when recovery is slow and limited. For example, Lwi et al. [56] reported that auditory comprehension scores improved ∼7% between 18 and 24 months post‐stroke (∼1.2% per month on average) and ∼7% between 60 and 120 months post‐stroke (∼0.12% per month on average). A nonlinear model may be less sensitive when there is little true recovery signal because transforming the data points inflates noise. In contrast, a linear model preserves the raw scale.

#### 3.3.2. Self‐Rated Scores

Within participants, no effect of age was observed on recovery score for any of the four self‐rated language skills (speaking, understanding, reading and writing), nor on the modality mean, in 559 participants after controlling for left and right hemisphere lesion size, amount of education, sex and handedness. No interactions were observed between age and any other variables.

## 4. Discussion

This study demonstrates a disadvantage of older age on language scores from multiple tasks, using data from 749 participants with a wide range of stroke severity and language abilities. Although the effect sizes were small, they were remarkably consistent across a wide range of left hemisphere lesion sizes, including participants with negligible damage (< 1 cm^3^) and no, or minimal language impairments post‐stroke. This pattern of response is therefore most likely to be explained by the impact of normal healthy ageing, rather than a consequence of the stroke. The novelty of these effects lies in how they vary with language task and participant characteristics. Specifically, we illustrate where normal ageing processes are heightened by post‐stroke effects. The following discussion is organised around the three study questions (A–C), followed by an examination of the study implications, limitations and future directions.

### 4.1. Which Language Tasks Are Most Sensitive to Age and Why?

As hypothesised, the strongest age‐related effects were found on expressive language tasks, particularly nonword repetition, semantic fluency and word repetition (Figure 3). However, contrary to previous studies [4, 40], age effects were more pronounced on comprehension tasks than on object naming, spoken picture description and letter fluency. These task‐specific differences likely reflect the varying cognitive and sensory demands of each task. Executive functions are particularly important for verbal fluency [57] because they require participants to generate multiple words, confined to a specific rubric (e.g., animals), while simultaneously inhibiting inappropriate competitors and retaining items already named to prevent perseveration. The stronger age effect we observe for semantic compared to letter fluency (Figure 3) is consistent with prior studies showing that lexical access speed has a greater effect on semantic than letter fluency in older participants [58]. In addition, letter fluency is a less familiar and more challenging task for both older and younger participants [59, 60], with higher intersubject variance washing out more subtle effects of age.

Even though we controlled for self‐reported hearing status, participants may have had mild undiagnosed/unperceived sensory hearing loss, common in older age irrespective of stroke. This will have influenced four of the tasks showing the strongest age effects (Figure 3): nonword repetition, word repetition, spoken word comprehension and spoken sentence comprehension. Nonword repetition and comprehension tasks also impose high demands on auditory memory and executive processing [61, 62]. In both spoken and written comprehension tasks, for example, participants must retain representations of speech in memory, while analysing pictorial information to match this auditory memory with the correct visual input.

There were some apparent contradictions, where age effects were observed on one task, but not another that tapped the same function. For example, age effects were strong for word repetition, but weaker for complex word repetition; similarly, they were strong for spoken word and sentence comprehension, but weaker for paragraph comprehension. We propose three possible explanations. First, tasks using simpler and shorter words (e.g., ‘vine’ in simple word repetition versus ‘defrosted’ in complex word repetition) activate denser phonological neighbourhoods, making them more susceptible to errors [63] and more sensitive to age‐related declines in cognitive and auditory–phonological processing, such as inhibition, auditory precision, processing speed and working‐memory capacity [64, 65]. Second, spoken paragraph comprehension involves understanding short stories, which may be less prone to age effects, as they (i) provide context, which aids responses (unlike in single‐word comprehension), and (ii) use simpler syntax (compared to the more linguistically complex stimuli in sentence comprehension). Third, complex word repetition and spoken paragraph comprehension had fewer trials (3 and 4, respectively), and therefore lacked the sensitivity needed to detect age‐related variability. Likewise, the absence of an age effect on the CAT digit span task may reflect insufficient trials to capture previously reported age‐related working memory decline [66].

Interestingly, age effects were weaker for object naming and spoken picture description, despite the known decline in word retrieval with age [62, 67] and the increased occurrence of ‘tip‐of‐the‐tongue’ states [68, 69]. This disadvantage may have been mitigated by larger vocabulary size in older adults [70, 71], which could benefit performance on both object naming and picture description tasks.

### 4.2. How the Effect of Age at Stroke Onset Is Modified by Other Key Variables

The disadvantage of older age at stroke was more pronounced in participants with large left hemisphere lesions and more severe initial symptoms. This novel finding clearly aligns with the notion that greater brain damage accelerates ‘brain age’ [36, 72, 73]. Specifically, larger lesions limit the residual neural capacity available to support language function and recovery. Severe aphasic symptoms compound these biological limitations and the disadvantage of ageing, as they impair learning ability and lessen the effects of language therapy, regardless of age [20, 74–77]. The greater disadvantage of older age in these participants may therefore reflect their reduced ability to recruit residual neural capacity compared to younger participants.

We also observed that the compounded challenge imposed by the combination of large lesions, severe initial severity and older age overrode the benefit of education on naming and picture description scores [55]. These results align with those of Aki Ö et al. [10], who found that older age reduced the benefit of more education on phonemic fluency. However, this observation is not consistent with the finding that the disadvantage of older age was reduced (as opposed to lost) in participants with more education [13].

### 4.3. The Effect of Age at Stroke Onset on the Degree of Recovery

No significant effect of older age on language recovery was observed on (i) long‐term CAT scores or (ii) longitudinal (within‐subject) change in self‐reported abilities between 1 week and 1 year post‐stroke. The absence of evidence that recovery is better in younger participants is consistent with 10 of the 18 prior studies that also found no age‐related impact on language recovery (Table S1).

## 5. Clinical Implications

Our findings highlight the sensitivity of the CAT to age at stroke onset, reflecting the additive combination of normal ageing processes with post‐stroke effects. Interpreting the severity of assessment scores should therefore be made with reference to an individual’s age and the factors associated with age, such as sensory impairments—as has been suggested for other aphasia assessments [78, 79]. The task‐dependent differences we observe also highlight which tasks are most sensitive to age in assessments—which could be essential for differential diagnosis, and selecting interventions. For older adults with larger/more severe strokes, age effects are more pronounced. It is paramount that these patients are not deprioritised for therapy on the basis of their age, particularly since we found that age did not have a significant effect on their recovery capacity. Rather, the finding that age effects are heightened when strokes are more severe should prompt earlier identification of those adults at greater risk, and encourage proactive planning and mitigation. Indeed, this may already be the case. Further studies are still required to investigate age effects on therapy provision or response and to assess the impact of varying therapy provision (including amount, intensity, timing and type) in older adults.

## 6. Study Limitations and Future Directions

The retrospective measurement of initial severity depends on participants’ memories, and is therefore subject to inaccuracy or bias. This recall bias could be greater in older compared to younger adults; however, it was not possible to assess whether this was the case in the current study, nor whether severity was more likely to be over‐ or under‐reported. The sample may also have been skewed towards patients with mild abilities, if the patients who were excluded due to missing retrospective scores were those who were too severe to remember the details of their early post‐stroke abilities. Future studies would benefit from assessing initial severity, in real time, using both standardised tests and patient‐reported outcome measures.

Hearing and vision abilities were also self‐reported, and thus potentially under‐reported, particularly amongst the older participants in the sample. This would result in underestimating their effect and potentially overestimating the age effect. Future studies could use instrumental measures like audiometry to detect subtle, undetected age‐related sensory changes that may impact language scores in participants with and without aphasia.

We were not able to control for all variables that might be influencing aphasia outcomes and recovery, for example, medical factors (e.g., diabetes, hypertension), speech therapy and brain age. The latter is particularly important given that brain age, derived from nonlesioned brain tissue, has been shown to explain more variance in aphasia outcomes than chronological age [39, 80]. Including these variables would elucidate how chronological age depends on brain age, cognitive function [30] and other factors.

Our interpretation that the small effects of age likely reflect healthy ageing processes rests on the assumption that participants with negligible lesion damage and no/minimal aphasia are akin to ‘healthy controls’. These participants were all still diagnosed with a stroke, and may have other non‐language impairments or accelerated cognitive decline, which could explain the observed effects. Future studies including healthy controls are needed to disentangle the impact of healthy versus accelerated ageing.

Although we used two different, within‐subject recovery measures, both had limitations. The first measured recovery as the difference between participant‐reported initial severity and CAT scores, which may not be sensitive to an effect of age on early recovery, because our ‘time post‐stroke’ variable spans a very wide range (1 month to 42 years). As a result, it likely amalgamates two competing effects: recovery, which tends to improve outcomes over time, and ageing, which is associated with gradual decline even in healthy adults. These opposing influences may complicate interpretation of time‐related changes post‐stroke, particularly when a long range of time post‐stroke is used. The second recovery measure used participant reports of language change during the first year post‐stroke, but may be too subjective and subtle to detect ability change. Additionally, by using a proportional recovery score, there could be bias towards participants with Mild initial severity—because less improvement is required for them to recover to normal (compared to those with Severe initial severity, in whom more improvement was required for full recovery to normal).

Finally, the median age at stroke (57 years) in this study’s sample was notably younger than the average age at stroke (71 years) in the United Kingdom in 2016 [1]. This bias is expected given the greater barriers to participating in research for older participants, e.g., more comorbidities and worse mobility [81, 82]. The ‘younger’ and ‘older’ threshold was based on median age (57 years) to ensure sufficient numbers in each subgroup, and not for any clinical labelling of individuals. This resulted in a wide age range in the ‘older’ subgroup (57–98 years, mean = 68 years). There is no definitive age at which a person is considered ‘older’, and the health and social care system rightly takes more aspects of an individual into account than just their chronological age, which might more accurately reflect their care needs, such as frailty [83]. Further studies are needed to investigate how age influences aphasia outcomes and recovery amongst the ‘older old’, and the extent to which these reflect normal ageing processes or post‐stroke effects. Extending the sample to include more participants in the oldest age range would also improve representativeness for aphasia prognosis models.

## 7. Conclusions

This study shows that participants who were older when they had a stroke performed worse on a range of language tasks from the CAT compared to participants who were younger when they had a stroke. This disadvantage of older age was found to prevail independent of left hemisphere lesion size and initial severity, likely reflecting normal ageing processes. However, the effect of older age was noticeably worse amongst participants with large left hemisphere lesions and severe initial aphasia, suggesting normal ageing effects are exacerbated by stroke damage. Older stroke survivors are therefore at risk of worse outcomes, particularly when strokes are large or severe. Our results also demonstrate the importance of interpreting assessment scores with consideration of age, and specifically age‐related cognitive and sensory impairments. Furthermore, recognising the predictive value of age at stroke (independent of that of lesion size and initial severity) motivates the inclusion of age in prognostic models of aphasia outcomes and recovery.

## Author Contributions

Study conception and design: S.M.R. and C.J.P. Data collection: S.M.R., R.M.B, S.A., H.W. and K.L. Data analyses: S.M.R. Manuscript drafting and preparation: S.M.R. and C.J.P. Manuscript revision and intellectual input: R.M.B., T.M.H.H., S.A., H.W., K.L., A.P.L., D.W.G. and C.J.P. Project supervision and funding acquisition: C.J.P.

## Funding

This work was funded by Wellcome [203147/Z/16/Z, 205103/Z/16/Z and 224562/Z/21/Z to C.J.P.]; the Medical Research Council [MR/M023672/1 to C.J.P]; and the Stroke Association [TSA 2014/02 to C.J.P. and D.W.G.]. A.P.L. was funded by The National Institute for Health and Care Research Professorship award [RP‐2015‐06‐012].

## Conflicts of Interest

The authors declare no conflicts of interest.

## Supporting Information

Additional supporting information can be found online in the Supporting Information section.

## Supporting information


**Supporting Information** A supporting Information file is included with this submission, which contains Tables S1–S5. Tables S1–S3 summarise prior studies of chronological age on aphasia outcomes and recovery, which are discussed in more detail in the Introduction. Tables S4 and S5 provide details of lesion size for the various subgroups in this study.

## Data Availability

The data that support these findings can be requested from the corresponding author.
