# Supplementary material for: The Effect of Age on Post‐Stroke Language Outcomes
Source: J Aging Res. 2026 Jun 23;2026:7040010. doi: 10.1155/jare/7040010 (PMC13290665; doi:10.1155/jare/7040010)
Supplement: Supplementary file 1 — Supporting Information A supporting Information file is included with this submission, which contains Tables S1–S5. Tables S1–S3 summarise prior studies of chronological age on aphasia outcomes and recovery, which are discussed in more detail in the Introduction. Tables S4 and S5 provide details of lesion size for the various subgroups in this study. [file JARE-2026-7040010-s001.docx]

**Table S1.** Literature investigating the influence of chronological age on (A) aphasia outcomes and (B) recovery, grouped by (1) significant or (2) non-significant effects.

| **Study** | **N** | **Outcome** | **Effect size** | **LESION** | **DEM** | **COG** | **CLIN** | **TPS** |
| --- | --- | --- | --- | --- | --- | --- | --- | --- |
| **A1: Significant age effects on aphasia outcome** | | | | | | | | |
| Nakagawa et al. (2019) | 121 | SLTA | NR | Y | Y | N | Y | N |
| Dresang et al. (2022) | 17 | WAB-AQ | -0.77 | Y | N | Y | Y | N |
| Wilson et al. (2023) | 334 | QAB | -0.03 | Y | N | N | N | Y |
| Naess et al. (2009) | 195 | SSS | NR | N | Y | N | Y | N |
| El Hachioui et al. (2013) | 147 | ASRS | 0.94 | N | Y | N | Y | N |
| Shin et al. (2022) | 4443 | K-FAST | NR | N | N | N | N | N |
| **A2: No significant age effects on aphasia outcome** | | | | | | | | |
| Basilakos et al. (2019) | 35 | WAB-AQ | n/a | Y | Y | N | N | Y |
| Gadson et al. (2022) | 81 | WAB-AQ | n/a | Y | N | N | N | Y |
| Johnson et al. (2022) | 106 | WAB-AQ | n.a | Y | N | Y | N | Y |
| Keenan and Brassell (1974) | 39 | 3-point scale | n/a | N | N | N | N | N |
| de Riesthal and Wertz (2004) | 34 | PICA & RFP | n/a | N | N | N | N | N |
| Oliveira and Damasceno (2009) | 37 | Improved vs not | n/a | N | N | N | N | N |
| **B1: Significant age effects on aphasia recovery** | | | | | | | | |
| Laska et al. (2001) | 119 | Grunntest & ANELT | NR | Y | Y | N | Y | N |
| Kertesz & McCabe (1977) | 93 | WAB-AQ | -0.240 | N | Y | N | Y | N |
| Holland et al. (1989) | 50 | WAB-AQ | -0.11 | N | Y | N | Y | Y |
| Kastrau et al. (2005) | 14 | AATB | NR | N | Y | N | Y | N |
| Kristinsson et al. (2023) | 107 | PNT | -0.20 | N | N | Y | N | Y |
| Johnson et al. (2019) | 39 | WAB-AQ | -1.51 | N | N | N | N | N |
| Shin et al. (2022) | 4443 | K-FAST | NR | N | N | N | N | N |
| **B2: No significant age effects on aphasia recovery** | | | | | | | | |
| Pedersen et al. (1995) | 881 | SSS Aphasia scale | n/a | Y | Y | N | Y | N |
| Lazar et al. (2008) | 22 | WAB & BDAE | n/a | Y | Y | N | Y | N |
| Pedersen et al. (2004) | 270 | WAB-AQ | n/a | Y | Y | N | N | N |
| Basilakos et al. (2019) | 35 | WAB-AQ | n/a | Y | N | N | Y | N |
| Kristinsson et al. (2022) | 30 | BEST-2 | n/a | Y | N | N | N | Y |
| Wilson et al. (2023) | 121 | QAB | n/a | Y | N | N | N | N |
| Pickersgill & Lincoln (1983) | 56 | PICA | n/a | N | Y | N | Y | N |
| Inatomi et al. (2008) | 121 | NIHSS | n/a | N | N | Y | N | N |
| Lendrem and Lincoln (1985) | 52 | PICA | n/a | N | N | N | N | N |
| Seniow et al. (2009) | 78 | BDAE | n/a | N | N | N | N | N |

**Legend:** Summary of studies that report the effect of age on (A) aphasia outcome and (B) recovery, grouped according to whether the effects were (1) significant or (2) not. NR = not reported. The columns indicate whether each study controlled for key methodological factors: LESION (lesion size, volume, extent, or location), DEM (demographic variables such as sex, education, race/ethnicity, income, or handedness), COG (non-linguistic cognitive factors or cognitive reserve), CLIN (clinical severity or stroke-related factors such as NIHSS, stroke type, comorbidities), and TPS (time post-stroke). Additional covariates unique to individual studies are listed in Table S2 below. Abbreviations for outcome measures are listed in Table S3 below.

| \| **Table S2: Unique Study-Specific Covariates Not Included in Table S1** \| \| \| \| \| \| --- \| --- \| --- \| --- \| --- \| \| **Study** \| **Gp** \| **N** \| **Outcome** \| **Unique Covariate(s)** \| \| **Dresang et al. (2022)** \| A1 \| 17 \| WAB-AQ \| BDNF genotype \| \| **Nakagawa et al. (2019)** \| A1 \| 121 \| SLTA \| Linguistic component scores \| \| **Basilakos et al. (2019)** \| A2 \| 35 \| WAB-AQ \| Fazekas score *(white matter disease severity measure)* \| \| **Gadson et al. (2022)** \| A2 \| 81 \| WAB-AQ \| Race; Income; Race × Lesion size interaction; Race × White matter disease interaction \| \| **Johnson et al. (2019)** \| B1 \| 39 \| WAB-AQ \| Antidepressant use; Exercise \| \| **Shin et al. (2022)** \| B1 \| 4443 \| K-FAST \| BMI; Smoking status; Alcohol use \| \| **Inatomi et al. (2008)** \| B2 \| 121 \| NIHSS \| Hypercholesterolemia; Hypertension; Diabetes; Smoking; History of stroke \|   Gp = Grouping in Table S1. A1 = Significant effect of age on outcome. A2 = Non-significant effect of age on outcome, B1 = Significant effect of age on recovery. B2 = nonsignificant effect of age on recovery.  **Table S3: Abbreviations for Outcomes in Tables S1 and S2** | | |
| --- | --- | --- | --- | --- | --- | --- | --- | --- | --- | --- | --- | --- | --- | --- | --- | --- | --- | --- | --- | --- | --- | --- | --- | --- | --- | --- | --- | --- | --- | --- | --- | --- | --- | --- | --- | --- | --- | --- | --- | --- | --- | --- | --- | --- | --- | --- | --- |
| **Abbreviation** | **Test name** |  |
| **AATB** | Aachen Aphasia Testing Battery |  |
| **ANELT** | Amsterdam–Nijmegen Everyday Language Test |  |
| **ASRS** | Aphasia Severity Rating Scale |  |
| **BDAE** | Boston Diagnostic Aphasia Examination |  |
| **BEST-2** | Bedside Evaluation Screening Test – Second Edition |  |
| **K-FAST** | Korean–Frenchay Aphasia Screening Test |  |
| **NIHSS** | National Institutes of Health Stroke Scale |  |
| **PICA** | Porch Index of Communicative Ability |  |
| **PNT** | Philadelphia Naming Test |  |
| **QAB** | Quick Aphasia Battery |  |
| **RFP** | Rating of Functional Performance |  |
| **SLTA** | Standard Language Test of Aphasia |  |
| **SSS** | Scandinavian Stroke Scale |  |
| **WAB-AQ** | Western Aphasia Battery – Aphasia Quotient |  |

**Table S4.** Mean and range of Left hemisphere lesion size within each lesion and severity group, in 572 participants (illustrated in Figure 1).

| **Lesion group** | **Initial severity** | **Younger** | | | **Older** | | |
| --- | --- | --- | --- | --- | --- | --- | --- |
|  |  | N | Mean | Range | N | Mean | Range |
| LH cm^3^: smaller | Severe | 30 | 0.66 | 0 to 3.14 | 14 | 0.98 | 0 to 2.26 |
|  | Moderate | 29 | 0.29 | 0 to 1.91 | 27 | 0.95 | 0 to 3.18 |
|  | Mild | 89 | 0.38 | 0 to 3.31 | 68 | 0.37 | 0 to 3.12 |
|  | Normal | 53 | 0.20 | 0 to 2.17 | 64 | 0.44 | 0 to 3.33 |
| LH cm^3^: larger | Severe | 49 | 34.66 | 8.98 to 68.38 | 48 | 35.12 | 8.51 to 65.06 |
|  | Moderate | 17 | 33.05 | 8.62 to 88.58 | 22 | 34.09 | 8.54 to 81.45 |
|  | Mild | 16 | 45.45 | 11.52 to 92.02 | 35 | 32.30 | 8.54 to 99.94 |
|  | Normal | 1 | 46.86 | 46.86 | 10 | 24.14 | 9.29 to 48.34 |

**Table S5.** Mean and range of Left hemisphere lesion size within each lesion and severity group, in 720 participants (illustrated in Figure 3).

| **Lesion group** | **Initial severity** | **Younger** | | | **Older** | | |
| --- | --- | --- | --- | --- | --- | --- | --- |
|  |  | N | Mean | Range | N | Mean | Range |
| LH: <1 cm^3^ | Normal | 48 | 0.036 | 0 to 0.848 | 51 | 0.035 | 0 to 0.89 |
|  | Mild | 75 | 0.054 | 0 to 0.992 | 56 | 0.053 | 0 to 0.85 |
|  | Severe | 46 | 0.087 | 0 to 0.960 | 22 | 0.080 | 0 to 0.94 |
| LH: 1 to 50 cm^3^ | Normal | 11 | 7.70 | 1.42 to 46.86 | 32 | 7.68 | 1.02 to 31.22 |
|  | Mild | 35 | 9.54 | 1.17 to 48.62 | 49 | 9.28 | 1.06 to 31.50 |
|  | Severe | 74 | 16.82 | 1.21 to 44.34 | 81 | 16.70 | 1.10 to 44.43 |
| LH: >50 cm^3^ | Normal | n/a | n/a | n/a | n/a | n/a | n/a |
|  | Mild | 7 | 96.39 | 62.16 to 182.52 | 8 | 86.84 | 51.11 to 171.08 |
|  | Severe | 66 | 117.70 | 50.08 to 225.34 | 59 | 112.51 | 50.49 to 235.12 |

**Legend:** The lesion size groups were determined by the size of the left hemisphere lesion, irrespective of the size of the right hemisphere lesion. The ‘Severe’ initial severity group includes participants with Moderate initial severity. The age group threshold was determined using the median of the full sample: Younger = up to 57.88 years; Older = 57.93 years and above. 29 participants were removed from the full sample to match the groups for left hemisphere lesion size.
